# The negative effects of anterior cruciate ligament injury during stroke rehabilitation

**DOI:** 10.12669/pjms.35.6.974

**Published:** 2019

**Authors:** Nawaf Alzamel, Ahmad Zaheer Qureshi, Farooq Azam Rathore, Sami Ullah

**Affiliations:** 1 Nawaf Alzamel, AlMaarefa University (MU), College of Medicine, Riyadh, Saudi Arabia; 2 Ahmad Zaheer Qureshi, Department of Physical Medicine & Rehabilitation, King Fahad Medical City, Riyadh, Saudi Arabia; 3 Farooq Azam Rathore, Department of Rehabilitation Medicine, PNS Shifa Hospital, Karachi, Pakistan; 4 Sami Ullah, Department of Physical Medicine & Rehabilitation, King Fahad Medical City, Riyadh, Saudi Arabia

**Keywords:** Anterior Cruciate Ligament, Complications, Length of stay, Musculoskeletal Diseases, Stroke Rehabilitation

## Abstract

Musculoskeletal problems are commonly reported after stroke resulting in abnormal gait biomechanics, pain, and limitation in performing activities of daily living. Anterior circulate ligament is the most frequently injured knee ligament accounting for approximately 50% of all ligament injuries; however, post stroke anterior cruciate ligament injury is rarely reported. We present a case of a 58-year-old female admitted for inpatient stoke rehabilitation after a left middle cerebral artery stroke. After gaining considerable functional recovery, she was planned to be discharged in two weeks’ time when she tripped resulting in a torn anterior cruciate ligament on the hemiperetic side. This resulted in increase in the length of stay and loss of functional gains. We discuss the possible mechanisms and the management plan. Patients with stroke should be monitored for musculoskeletal complications and preventive strategies should be devised to protect from possible ligamentous injuries of the knees.

## INTRODUCTION

Stroke is a major cause of disability which has considerable effects on patients and their families. More than 80% of patients with stroke have residual motor weakness and require rehabilitation.^1^,^2^ Musculoskeletal problems are commonly reported after stroke resulting in improper gait biomechanics, development of pain, and limitation in performing activities of daily living.^3^ Anterior Cruciate Ligament (ACL) is the most frequently injured knee ligament and accounts for approximately 50% of all ligament injuries; however, post stroke ACL injury is rarely reported.^4^ Though ACL dysfunction is not a direct outcome of stroke; its association with these sensory motor impairments post stroke is a unique entity. The aim of this case report is to highlight the negative impact of ACL injury outcomes of stroke.

## CASE REPORT

Informed consent was taken from the patient and the case report was approved by institutional review board. A 58 years old female with diabetes mellitus, hypertension and dyslipidemia was admitted for inpatient rehabilitation after two weeks of left middle cerebral artery (MCA) stroke. She had a body mass index of 28.9 kg/m^2^. Her main impairments at admission were non-fluent aphasia and right hemiparesis. Her non-verbal communication was intact, and she had mild limitation in initiation and planning. There were no cranial nerve deficits and she did not have any visual impairment. The neurological examination of the left upper and lower limb was unremarkable. On the right upper and lower extremities, muscle tone was normal, sensations were intact and passive range of motion was within normal limits in all joints. Muscle power in the right upper limb was 2/5 in shoulder muscles, elbow flexors, wrist flexors and finger flexors with a weak grip. In the right lower extremity, her muscle strength was 3/5 in hip flexors, knee extensors and ankle planter and dorsiflexors. Reflexes were exaggerated on the affected side with ankle clonus. The coordination could not be assessed in the right upper limb due to her weakness; however, her coordination and proprioception was intact in the right lower extremity. Her Morse fall risk assessment score was 55 at admission and her Functional Independence Measure (FIM) score was 48. She required moderate assistance in grooming and dressing upper body, and maximum assistance in dressing lower body, bathing, toileting and transfers. She had a good static and fair dynamic sitting balance; while her standing balance was poor. She was enrolled in an intensive in-patient stroke rehabilitation program including three hours per day of physical therapy, occupational therapy and speech therapy for five days a week. The plan was a short stay admission for ten days and the functional goals of rehabilitation were to be modified independent in all activities of daily living and transfers, and to be able to walk indoor with a cane. Long term goal was to improve her participation and to enable her to return to her previous role as a homemaker.

Her weight was a barrier in performing her activities; however, she made considerable functional gains in her mobility after five therapy sessions and was able to sit to stand with minimal assistance, and could do pivot transfers with maximum assistance. She was able to ambulate 25 feet using quad cane without the need of ankle foot orthosis and could climb five steps of stairs using hand rails. She was permitted to ambulate with caregiver with contact guarding after therapy sessions. After working hours, the patient was ambulating in the garden with her caregiver when she walked over an uneven surface and suddenly felt severe pain in the knee. On examination, there was diffuse knee swelling with tenderness on the medical aspect of knee and limited active and passive range of motion. Special tests for knee instability including anterior and posterior drawer signs, Lachman’s test and stress tests for medial and lateral collateral ligaments were unremarkable. MacMurray test was inconclusive due to severe pain. The low molecular weight heparin was withheld due to concerns of hematoma, and patient was started on oral Meloxicam 15 mg once daily, Paracetamol1 gm thrice daily orally, Diclofenac Sodium gel local application four times daily with compression garments and icing. An X-ray of the knee ruled out any bony or joint abnormality. Magnetic Resonance Imaging (MRI) of the knee revealed a tear at the posterior horn root junction with meniscal extrusion. There was a high-grade partial tear of the ACL associated with a large complex joint effusion likely due to hemarthrosis related to the trauma. In consultation with orthopedics, conservative therapy was planned. Comprehensive rehabilitation was resumed and patient was started on range of motion exercises, strengthening of knee extensors, stretching of hamstrings and proprioceptive training. Gait training was resumed with full weight bearing as tolerated and she received two hours of therapy divided in two sessions per day. The length of stay of patient was extended two more weeks from the intended date of discharge. At discharge from rehabilitation, she had modified independence in bed mobility, could sit to stand and transfer under supervision. We tried to train her to walk with a simple cane but her distance was limited to 50 feet due to knee pain and apprehension of fall. With a walker, she was able to walk for 150 feet with supervision and preferred to use knee immobilizer even though it was not recommended by orthopedics. She had a total FIM gain of 32 points with a FIM score of 80 at discharge. Her Morse fall risk assessment score decreased by 15 points at discharge.

## DISCUSSION

Different upper and lower limb musculoskeletal problems are associated with stroke.^2^ These conditions can contribute to the abnormal biomechanics, dependency in activities of daily living, difficulty in ambulation and a high risk for falls (Table-I).^2^-^7^ This case, who was otherwise ready to be discharged after a short inpatient admission, ended up staying longer and had a decline in function due to a preventable knee injury. This was accounted towards cost of care, patient satisfaction and functional impacts.

**Table I:** Common painful and disabling musculoskeletal conditions after stroke.^2^-^7^

Body Area	Joint Involved	Condition
Upper Limbs	Shoulder	Gleno-Humeral Subluxation Shoulder Impingement Adhesive Capsulitis
Lower Limbs	Hip	Post-stroke hip fractures Greater trochanteric pain syndrome Gluteus Mediustendinosis Hip external rotator Muscles strain Heterotopic ossification Myofascial pain Syndrome
	Knee	Stiff knee gait Genu recurvatum
	Ankle	Varus deformity of foot Claw toes Persistent extension of the great toe

Köseoğlu and Colleagues evaluated the impact of lower extremity pain on clinical variables, and health-related quality of life in one hundred and eighty-five patients with stroke.^2^ Using self-reported measures of pain, the common causes of lower extremity pain documented were osteoarthritis, central neuropathic pain, mixed pain, low back pain associated with leg pain, greater trochanteric pain syndrome, prior hip fracture, heterotopic ossification, developmental hip luxation, hallux valgus, and malignancy. The pain in majority of their cases was classified as musculoskeletal, moderate in intensity, involving knee and started after the stroke. They suggested that the lower extremity pain can negatively affect rehabilitation outcomes after stroke and result in a poor health related quality of life in stroke survivors.^2^

ACL contains mechanoreceptors that can identify changes in tension, speed, acceleration, direction of movement, and the situation of the knee joint.^8^ ACL plays a major role in the knee biomechanics and stability during standing and ambulation. The major mechanical function of the ACL is to prevent excessive anterior tibial translation in different degrees of flexion. During knee flexion, the anteromedial band of the ligament mainly prevents anterior tibial translation, whereas the posterolateral band contributes mostly to stability in extension.^9^ The ACL also prevents hyperextension of the knee, limits excessive tibial rotation (internal rotation more than the external rotation), and is a secondary restraint to both valgus and varus stresses. ACL injury results in decrease in proprioception. This becomes more important for a patient with stroke. The knee biomechanics of a patient with stroke are already compromised due to a combination of muscle weakness, spasticity and decreased proprioception. An ACL tear is likely to aggravate the ambulation dysfunction and negatively affect the rehabilitation protocol as described in this case report. The ACL tear resulted in extra therapy sessions, modification of the rehabilitation protocol and increased length of stay in hospital. The most possible risk factors for ACL injury in our patient are listed in Table-II.^10^ Additional non-patient factors which led to the possible mechanism of injury in our patient were mostly preventable. It included walking unsupervised on an uneven surface with an inappropriate foot wear. Extensive education with the patient and caregivers could have prevented the patient from sustaining an ACL injury by improving compliance to instructions provided by therapists. A risk stratification protocol may be established to identify cases at risk; which requires extensive review of similar cases. Use of functional knee brace do not sufficiently restore normal biomechanics to patients with ACL injury and currently no brace has been validated to replicate the force dynamics of the native ACL.^11^ Hence it was not recommended in our patient; however, it may have helped her by providing proprioceptive feedback.

**Table II:** Risk factors for non-impact ACL injury.

For non-impact ACL Injury^10^	Relevant to stroke	Relevant to case report
Genetics		
Female Gender		Female Gender
Hormonal Concentration	Hormonal changes occur after stroke	
Increased knee joint laxity	Laxity may be evident in patients with flaccidity especially during initial phase of motor recovery after stroke.	
Decreased intercondylar notch width		
Increased posterior tibial slope		
Neurocognitive factors	Slow reaction time Processing speed Impulsivity Poor insight Impaired judgment Visuo-spatial impairment Neglect	Slow reaction time secondary to left MCA stroke.
Neuromuscular factors	Weakness Proprioceptive impairment Spasticity and Limb dystonia Associated reaction Co-contraction Ankle Clonus Imbalance Incoordination Fatigue	Weakness Imbalance Ankle Clonus
Prior ACL injury		
Extrinsic factors	Ground surface, footwear	Uneven surface (garden) Wearing inappropriate shoes (open shoes)

There is only one documented case report of ACL tear in a 17-year-old patient who sustained an ACL injury nine years after her stroke while playing soccer, involving the side affected by stroke earlier.^4^ MRI revealed right ACL rupture with potential meniscal involvement. She underwent ACL reconstruction the following month and post-operative rehabilitation plan consisted of range of motion exercises, progressing to strengthening exercises of lower limbs and aquatic therapy. In order to improve the limited knee extension, dynamic knee extensor brace was applied. She made good functional recovery over the next two months. Our patient has some similarities and differences from this case. The former case is of a young female who had a chronic stroke with minimal residual deficits and had a sports injury resulting in ACL tear nine years after stroke. In contrast, our patient is a middle-aged obese woman who had a non-contact ACL injury during her rehabilitation within one month of stroke. A systemic review reports that outcomes of ACL reconstruction in obese and overweight patients showed low knee function scores and are associated with developing arthritis.^12^ Additional risk factors predisposing to knee instability in our patient included weakness, clonus and poor balance; hence rendering further challenge to ACL reconstruction; however, this assumption is theoretical as outcomes of ACL reconstruction in neurologically impaired limb is not well-studied. The above reasons led to consider non-surgical treatment in our patient. It is arguable that treating a high grade partial ACL tear conservatively in an obese patient post stroke may predispose the patient to poor outcomes; however, more studies need to be done to establish a consensus in such situations. Smith et al, in their meta-analysis reported that literature suggests limited difference in clinical outcomes between ACL reconstruction and non-operative treatment, and an ACL rupture should receive non-operative treatment before surgical intervention is considered.^13^ Commonly, surgical approach is considered in physically demanding individual especially to resume pivotal sporting activities, as it is advocated that an early ACL reconstruction helps to restore the kinematics of the tibiofemoral joint, thus reducing the risk of joint instability.^14^ Since, there are no protocols for ACL rehabilitation in individuals with stroke, our program was similar to standard protocols for ACL rehabilitation, focusing on progressive resolution of impairments, focusing on pain, swelling, range of motion, weight bearing, gait, proprioceptive training and progressive strengthening followed by maximizing neuromuscular control. Post stroke neurological impairments were taken into consideration by modifying the program.

## CONCLUSION

Musculoskeletal disorders are common after stroke and can result in pain, disability and poor quality of life. ACL tear after stroke is rare and can negatively affect the rehabilitation outcomes. Patients with stroke should be monitored for musculoskeletal complications and preventive strategies should be devised to protect from possible ligamentous injuries of the knees.

### Authors’ Contributions:

**NZ:** Identified the case, plotted the initial manuscript and searched literature.

**AZQ & FAR:** Drafting of manuscript with additional literature review.

**SU & AZQ:** Critical review of manuscript and helped out in initial conceptualization.

**SU & NZ:** Final editing and revision of manuscript.
